# Effective dose 50 method as the minimal clinically important difference: Evidence from depression trials

**DOI:** 10.1016/j.jclinepi.2021.04.002

**Published:** 2021-09

**Authors:** Clarissa Bauer-Staeb, Daphne-Zacharenia Kounali, Nicky J. Welton, Emma Griffith, Nicola J. Wiles, Glyn Lewis, Julian J. Faraway, Katherine S. Button

**Affiliations:** a Department of Psychology, University of Bath, Bath, United Kingdom; b Bristol Medical School, University of Bristol, Bristol, United Kingdom; c Avon and Wiltshire Mental Health Partnership NHS Trust, Bath, United Kingdom; d Division of Psychiatry, University College London, London, United Kingdom; e Department of Mathematical Sciences, University of Bath, Bath, United Kingdom

**Keywords:** Minimal Clinically Important Difference, MCID, Primary Care, PHQ-9, GAD-7, Clinically Meaningful Change

## Abstract

**Objective:**

Previous research on the minimal clinically important difference (MCID) for depression and anxiety is based on population averages. The present study aimed to identify the MCID across the spectrum of baseline severity.

**Study Design and Settings:**

The present analysis used secondary data from 2 randomized controlled trials for depression (*n* = 1,122) to calibrate the Global Rating of Change with the PHQ–9 and GAD–7. The MCID was defined as a change in scores corresponding to a 50% probability of patients "feeling better", given their baseline severity, referred to as Effective Dose 50 (ED50).

**Results:**

MCID estimates depended on baseline severity and ranged from no change for very mild up to 14 points (52%) on the PHQ–9 and up to 10 points (48%) on the GAD–7 for very high severity. The average MCID estimates were 3.7 points (23%) and 3.3 (28%) for the PHQ–9 and GAD–7 respectively.

**Conclusion:**

The ED50 method generates MCID estimates across the spectrum of baseline severity, offering greater precision but at the cost of greater complexity relative to population average estimates. This has important implications for evaluations of treatments and clinical practice where users can use these results to tailor the MCID to specific populations according to baseline severities.

## 1 Introduction

Depression and anxiety are the most common mental health problems worldwide [1]. In the absence of objective tests, self-report questionnaires are frequently used to measure symptom change. However, uncertainty remains about how much change on these questionnaires is clinically meaningful. A first step toward conceptualizing clinically meaningful improvement has been to define minimal clinically important differences (MCID) – the smallest difference in scores that are of perceived benefit to patients [2]. Although various methods of estimating important differences on questionnaires exist, it is imperative to include patients’ perceptions to define clinically meaningful change [2], [3]–4], particularly where subjective experiences, such as depression, and anxiety, are targeted. Anchor–based approaches, which anchor questionnaire outcomes onto patient reports of subjective improvement, are truly patient-centered by incorporating the patients’ experiences [2].

Early work estimating the MCID using these methods for the Beck Depression Inventory-II demonstrated baseline dependency [5,6]. Patients with a higher baseline severity require larger changes to experience a subjective improvement. Various methods exist to address this problem (Supplementary Material A); however, 2 commonly used methods are the standardized mean differences amongst those who report slight improvements compared to those who feel the same or proportionate change - percentage change in symptoms relative to baseline [5], [6], [7], [8]–9]. Recent research has explored the MCID for depression and anxiety on the Patient Health Questionnaire (PHQ-9) and Generalized Anxiety Disorder Scale (GAD-7) [10], [11], [12]. Collectively, the research suggests that the MCID can be defined as approximately 20% improvement ,[5], [9], [12]. Although providing a good rule-of-thumb, they are unable to fully capture baseline dependency equally well across all patients – the 20% estimate applies less well to patients with lower baseline severity or patients with treatment resistant depression with higher baseline severity [5], [12]. This is substantiated by research demonstrating a 51% disagreement when comparing the 20% MCID to patient self-reported improvement [13]. Standardized effect sizes have been criticized for being difficult to interpret and providing little clinical information [14]. Given this, there is a need to further address baseline dependency when estimating the MCID. In light of the above, we present a novel approach to estimate a baseline-dependent MCID for widely used measures of depression and anxiety – the PHQ-9 and GAD-7 [10,11].

## 2 Methods

### 2.1 The sample

The present study used data from two, multi-center randomized controlled trials (RCTs): PANDA (What are the indications for prescribing ANtiDepressants that will leAd to a clinical benefit) and CoBalT. [15,16] PANDA (*n* = 653) compared sertraline vs. placebo in primary care patients where there was clinical equipoise about the benefits of antidepressant medication [15]. CoBalT (*n* = 469) compared cognitive behavioral therapy (CBT) as an adjunct to usual care (pharmacotherapy) to usual care alone, in primary care patients with treatment resistant depression [16]. The data was pooled across RCTs, resulting in more observations at each level of baseline severity and therefore increasing the precision of analyses. Data from all treatment arms was used as we assume a stability between change in symptoms and subjective improvement, irrespective of how the change in symptoms is brought about. Pooling data from both RCT and across treatment arms also increases the generalizability of the results.

### 2.2 The 9-item patient health questionnaire (PHQ-9) and the 7-Item generalized anxiety disorder scale (GAD-7)

The PHQ-9 and GAD-7 are self-report questionnaire assessing the severity of depression and anxiety symptoms over the past 2 weeks, respectively [10,11]. The range for the PHQ-9 is 0-27 and 0-21 on the GAD-7, with higher scores indicating greater symptom severity. The PHQ-9 and GAD-7 were completed at baseline, 2-, 6-, and 12-weeks in PANDA [15]. In CoBalT, the PHQ-9 was measured at baseline, 3-, 6-,9-, and 12-months whereas the GAD-7 was collected at baseline, 6- and 12-months [16].

### 2.3 Global rating of change (GRC)

Both PANDA and CoBalT included the 1-item GRC asking patients how they felt compared to when they were last seen [15], [16], [17], [18]. The GRC was measured at all follow-up time points. CoBalT patients could respond: “I feel better”, “I feel about the same”, and “I feel worse” [16]. In PANDA, patients could respond: “I feel a lot better”, “I feel slightly better”, “I feel about the same”, “I feel slightly worse”, and “I feel a lot worse” [15]. For all models, groups were dichotomized into *feeling better* and *not feeling better,* as the aim was to estimate the point at which patients experience an improvement. The category *not feeling better* consisted of patients who felt the same or worse.

### 2.4 Statistical analysis

An extensive methodological justification can be found in supplementary material A. All analyses were performed in the R statistical programming language [19].

#### 2.4.1 Modelling change across time

Change across multiple follow-ups was calculated from the previous timepoint (a rolling baseline), so at time *t* the change is: *x _(t-1)_ –* x_t,_ where *x_t_* is the follow-up score at timepoint *t*. Negative scores indicate deteriorations in symptoms whereas positive scores indicate improvements in symptoms.

To establish that the GRC is an appropriate anchor, Spearman rank correlation coefficients were estimated, examining the association between the categorical GRC and change scores. Correlation coefficients ≥0.30 have been deemed as appropriate [20]. This threshold was exceeded across studies and time points ranging from -0.32 to -0.52 (Supplementary Material B).

#### 2.4.2 Generalized additive mixed models (GAMM)

GAMMs provide a flexible approach to model complex, interacting relationships although maximizing model fit. A logistic GAMM was fitted, specifying the binary GRC (*better vs. not better*) as the outcome using the “mgcv” package [21]. Change in symptom scores and baseline severity were classed as predictors with an interaction term, given the established importance of baseline dependency [4,^5^,12]. Due to the repeated measurement, a random intercept was included for patients [15,16]. There is a natural variation in GRC responses between individuals – different patients will be more or less likely to respond feeling *better* or *not better* even when accounting for baseline severity and change*.* Random effects can account for the correlation between repeated observations of the same individual. To deal with the intrinsic correlation between change and baseline scores as well the bounded nature of the scales thin plate splines with a monotonicity constraint were used to model the combined effect of change and baseline severity on the response [21]. As the data were obtained from 2 separate studies and collected over multiple follow-up periods, a further model evaluated the effects of time and study by adding these as covariates. Model summaries and 95% confidence intervals can be found in Supplementary Material C and D, respectively.

#### 2.4.3 Effective dose 50 (ED50)

In the present study we applied the ED50 as a new method to estimate the MCID. ED50 is an interpretable and well-validated measure used in drug safety and pharmaceutical research to determine minimum thresholds for effective therapeutic doses [22]. Applied to the current context, the ED50 is the change in scores where there is a 50% probability of patients reporting *feeling better*. The ED50 has face validity as an MCID as it identifies the smallest point where a patient might be marginally more likely to *feel better* than *not*. Further face validity is added to the concept of using the ED50 as a MCID given that the lowest bound of response to treatment is often defined as a 50% improvement [23]. Here, this principle is applied to the subjective experience of improvement rather than the symptom measure itself. From the GAMMs, we predicted the probability of response and identified the change in scores associated with 50% probability of *feeling better*. A limit of 0 change was set, as it would be clinically unacceptable to classify symptom deteriorations as improved. The absolute MCIDs were converted to a percent change from baseline. The ED25 and ED75 - the point at which there is a 25% and 75% probability that the patient reports *feeling better* - were also calculated as interval estimates, providing an index of variability of *feeling better* under different clinically acceptable probabilities. Furthermore, the sensitivity, and specificity of the ED50 as well as the agreement between the MCID and patient-reported improvement were estimated.

#### 2.4.4 Standardized mean difference (SMD)

To allow for comparisons with more traditional methods, the crude and standardized mean difference between those “feeling slightly better” and those “feeling about the same” were examined in PANDA using the “TableOne” package [7], [8]–9,24]. These data were not available in CoBalT.

## 3 Results

### 3.1 Sample characteristics

Baseline sociodemographic and clinical characteristics of all patients recruited into the RCTs are presented in Table 1. Patients in PANDA had a lower clinical severity at baseline, with moderate symptoms of depression and mild anxiety. Patients in CoBalT had higher scores across all measures with severe depression and moderate anxiety scores. Table 2 shows the mean change associated with GRC responses, stratified by study and follow-up.

**Table 1:** Sociodemographic and clinical characteristics, stratified by study

	PANDA	CoBalT
*n*	*653*	*469*
Age (years)	39.70 (14.93)	49.59 (11.70)
Female	384 (59%)	339 (72%)
Whitea	579 (89%)	459 (98%)
Marital Status^a^		
*Married or living as married*	255 (39%)	248 (53%)
*Single*	296 (45%)	89 (19%)
*Separated, divorced, or widowed*	101 (15%)	132 (28%)
In paid employment^a^	433 (66%)	206 (44%)
Highest educational qualification^a^^,^^b^		
*A level, higher grade or above*	450 (69%)	217 (47%)
*GCSE, standard grade or other*	169 (26%)	130 (28%)
*No formal qualifications*	33 (5%)	116 (25%)
Financial difficulty^a^		
*Living comfortably or doing alright*	364 (56%)	167 (36%)
*Just about getting by*	204 (31%)	174 (37%)
*Finding it difficult or very difficult to make ends meet*	84 (13%)	128 (27%)
Number of life events in past 6 months	1.22 (1.19)	1.25 (1.15)
SF-12 mental health subscale	32.47 (11.04)	28.60 (9.14)
SF-12 physical health subscale	52.07 (9.70)	43.45 (13.47)
Patient Health Questionnaire-9	12.00 (5.80)	16.59 (5.67)
Generalized Anxiety Disorder Scale-7	9.43 (5.28)	11.75 (5.05)

a Data missing for one person in Panda.

b Data missing for six people in CoBalT.

**Table 2:** Mean change in outcome questionnaires, stratified by Global Rating of Change, study, and follow-up

			Baseline to Follow-up 1					Follow-up 1 to Follow-up 2					Follow-up 2 to Follow-up 3					Follow-up 3 to Follow-up 4				
		Global Rating of Change	Mean Baseline (SD)	*n*	%	Mean Change	*SD*	Mean Baseline (SD)	*n*	%	Mean Change	*SD*	Mean Baseline (SD**)**	*n*	**%**	Mean Change	*SD*	Mean Baseline (SD)	*n*	%	Mean Change (SD)	*SD*

Patient Health Questionnaire-9	*PANDA*	A lot better	11.89 (5.76)	35	6	6.51	*4.85*	10.04 (5.68)	110	21	5.11	*4.74*	8.25 (5.68)	118	23	3.14	*4.31*	-	-	-	**-**	-
		Slightly better		164	29	3.57	*4.52*		168	32	2.70	*4.46*		143	28	2.10	*3.55*		-	-	**-**	-
		About the same		291	51	0.95	*3.62*		172	32	0.42	*3.35*		174	34	0.22	*3.24*		-	-	**-**	-
		Slightly worse		63	11	-0.19	*3.58*		65	12	-1.80	*4.00*		66	13	-2.39	*4.79*		-	-	**-**	-
		A lot worse		15	3	-5.60	*4.93*		16	3	-4.75	*3.96*		17	3	-6.65	*5.44*		-	-	**-**	-
	*CoBalT*	Better	16.48 (5.69)	214	49	6.51	*6.00*	12.59 (6.12)	202	49	4.04	*5.40*	10.81 (6.88)	171	45	2.23	*4.96*	10.42 (6.74)	174	48	2.34	*5.03*
		Same		168	38	2.19	*5.10*		140	34	0.61	*5.17*		121	32	0.52	*4.89*		124	34	-0.38	*4.71*
		Worse		57	13	-1.70	*6.08*		69	17	-3.07	*6.28*		86	23	-3.42	*5.94*		62	17	-2.89	*6.26*
Generalized Anxiety Disorder Scale-7	*PANDA*	A lot better	9.27 (5.29)	35	6	6.00	*5.57*	7.79 (5.35)	109	21	4.34	*4.50*	6.16 (5.17)	117	23	2.16	*3.91*	-	-	-	-	*-*
		Slightly better		163	29	2.96	*4.55*		166	31	2.28	*3.69*		143	28	1.69	*3.71*		-	-	-	*-*
		About the same		292	51	0.59	*3.62*		171	32	0.58	*3.61*		172	33	-0.01	*3.01*		-	-	-	*-*
		Slightly worse		63	11	0.06	*4.26*		65	12	-1.09	*4.03*		66	13	-1.73	*3.71*		-	-	-	*-*
		A lot worse		15	3	-4.80	*4.28*		16	3	-4.25	*4.22*		17	3	-4.24	*4.96*		-	-	-	*-*
	*CoBalT*	Better	-	**-**	-	**-**	*-*	11.64 (5.02)	205	49	6.31	*5.21*	-	**-**	*-*	-	*-*	8.12 (5.86)	186	48	2.27	*4.39*
		Same		**-**	-	**-**	*-*		142	34	1.34	*4.15*		**-**	*-*	**-**	*-*		135	35	-0.22	*4.38*
		Worse		**-**	-	**-**	*-*		72	17	-0.68	*4.90*		**-**	*-*	**-**	*-*		65	17	-2.88	*4.48*

*Generalised Anxiety Disorder Scale-7 data was not collected at follow-up one and three. Baseline and change scores are derived from previous follow-up.

SD - Standard deviation

Data reported for patients with complete Global Rating of Change and change scores on each respective outcome questionnaires.

### 3.2 GAMM

We found statistically significant effects of study and time on the probability of feeling better. However, as might be expected, these made little to the MCID estimates and were therefore omitted from the final model for interpretability and generalizability. Of note, the effects of study on probability of feeling better appear to be driven by the differing baseline severities of the two samples at time point 1 due to their differing selection criteria. Combining the datasets is advantageous as it provides rich data across the distribution of baseline scores and the model produces a weighted average that accounts for the number of observations in each study.

### 3.3 ED50

Table 3 shows the ED estimates for both questionnaires. Across the PHQ–9 and GAD–7, patients with minimal symptoms at baseline need no change to have at least a 50% probability of feeling better; however, as severity increases the ED estimates increase in incremental steps. However, this is not a uniform, linear pattern, demonstrating the complexity of the effect change and baseline severity have on the probability of feeling better.

**Table 3:** The Minimal Clinically Important Difference at each level of baseline severity

		Patient Health Questionnaire -9				Generalized Anxiety Disorder Scale -7			
Baseline Score	Clinical Cut-Off	ED25	ED50	ED50 (%)	ED75	ED25	ED50	ED50 (%)	ED75

1	Minimal	0	0	0	1	0	0	0	N.A.
2		0	0	0	2	0	0	0	2
3		0	0	0	2	0	0	0	3
4		0	0	0	2	0	0	0	3
5	Mild	0	0	0	3	0	1	20	4
6		0	0	0	3	0	2	33	5
7		0	1	14	4	0	2	29	5
8		0	1	13	4	0	3	38	6
9		0	2	22	5	0	4	44	7
10	Moderate	0	3	30	5	0	4	40	7
11		0	3	27	6	0	5	45	8
12		0	4	33	6	1	5	42	8
13		0	4	31	7	2	6	46	9
14		1	5	36	7	2	6	43	9
15	Severe	1	5	33	8	3	7	47	10
16		2	5	31	9	3	7	44	11
17		2	6	35	9	4	8	47	11
18		3	7	39	10	5	8	44	12
19		3	7	37	11	5	9	47	12
20		4	8	40	12	6	10	50	13
21		4	9	43	13	6	10	48	13
22		5	10	45	14	-	-	-	-
23		6	11	48	14	-	-	-	-
24		7	11	46	15	-	-	-	-
25		7	12	48	16	-	-	-	-
26		8	13	50	17	-	-	-	-
27		9	14	52	18	-	-	-	-
Average across sample		1.2	3.7	23.3	6.4	1.0	3.3	28.0	6.1

ED25, effective dose 25; ED50, effective dose 50; ED75, effective dose 75; N.A, not available.

The ED50 score averaged over patients coincides with moderate depression (PHQ–9) and mild anxiety (GAD–7).However, there was a large range of MCID estimates, from 0 points (0%) up to 14 points (52%) on the PHQ-9, and up to 10 points (48%) on the GAD–7. Larger changes are needed on the GAD–7 than the PHQ-9 to feel *better*.

The models could not predict higher probabilities of feeling better amongst patients with very low baseline severity on the GAD–7, given the marginal ability to improve in symptoms. Patients would have to change more than is possible to obtain high probabilities of improvement. For clinical interpretation, equating these to 100% change is reasonable.

### 3.4 Sensitivity and specificity

Table 4 demonstrates that the ED50 estimates shows adequate sensitivity and specificity, providing a reasonable estimate for the smallest change in scores needed to *feel better.* The specificity of the ED50 was generally higher than the sensitivity and did not fall below 0.70, which could be deemed a clinically acceptable threshold. The disagreement between GRC and improvements based on the ED50 was 28.4% on the PHQ–9 and 28.9% on the GAD–7.

**Table 4:** Sensitivity and specificity of the Minimal Clinically Important Difference for the overall sample and stratified by study

	Patient Health Questionnaire -9		Generalized Anxiety Disorder Scale -7	
	Sensitivity	Specificity	Sensitivity	Specificity

Overall	0.65	0.77	0.67	0.75
PANDA	0.69	0.73	0.65	0.72
CoBalT	0.61	0.83	0.70	0.81

### 3.5 SMD

Table 5 shows the mean difference between those *feeling the same* and those *feeling slightly bette*r was ~ 2 points on both questionnaires. The SMD on the PHQ–9 was ~0.6 and ~0.5 on the GAD–7.

**Table 5:** Standardized Mean Difference based on subgroups of the Global Rating of Change, stratified by time in PANDA

	Feeling Slightly Better	Feeling the Same		
Patient Health Questionnaire -9	*Change (SD)*	*Change (SD)*	*Crude Difference*	*Standardized Mean Difference*

Baseline to Follow-up 1	3.57 (4.52)	0.95 (3.62)	2.62	0.64
Follow-up 1 to Follow-up 2	2.70 (4.46)	0.42 (3.35)	2.28	0.58
Follow-up 2 to Follow-up 3	2.10 (3.55)	0.22 (3.24)	1.88	0.55
Generalized Anxiety Disorder Scale-7				
Baseline to Follow-up 1	2.96 (4.55)	0.59 (3.62)	2.37	0.58
Follow-up 1 to Follow-up 2	2.28 (3.69)	0.58 (3.61)	1.7	0.47
Follow-up 2 to Follow-up 3	1.69 (3.71)	-0.01 (3.01)	1.7	0.50

SD, standard deviation

## 4 Discussion

A patient-centered approach was taken to estimate the MCID for widely used measures of depression and anxiety. The MCID was defined in a novel way as the change in scores that reflects at least a 50% probability that patients report *feeling better*. We produced MCID estimates stratified by severity scores, which increased with baseline severity in a non-linear manner, ranging from no change for very mild up to 14 points (52%) on the PHQ-9 and up to 10 points (48%) on the GAD–7 for high severity. Across the sample, the average MCID estimates were 3.7 points (23%) and 3.3 (28%) for the PHQ–9 and GAD–7 respectively. For comparative purposes, the (standardized) mean difference method was applied to PANDA yielding estimates of ~0.6 and ~0.5 for the PHQ–9 and GAD–7 respectively [7–9].

Previous research modelling proportionate change suggests the MCID is  ~ 20–30% improvement for moderately-severe populations for depression and anxiety respectively [5], [12]. Specifically, for patients of a moderate baseline severity a MCID of 21% change on the PHQ-9 and a 27% on the GAD–7 were previously reported, which translates into a 1.7- and 1.5-point improvement, respectively and standardized mean differences ~0.5 [12]. This is consistent with other medical fields where MCIDs defined as effect sizes range from 0.3–0.5 [5,9]. Primary care services providing psychological therapy for depression and anxiety in England currently a use a 6- and 4- point change for the PHQ–9 and the GAD–7 respectively to capture improvement, which are based on the Jacobsen and Traux’s Reliable Change Index [25,26].

The MCID is a concept, it is not mathematically defined. There are various methods by which it can be estimated, each with different modelling assumptions and inferential objectives, meaning any comparisons between estimates are indirect and crude. However, the flexibility of the present method allows different levels of the probability of response to be modelled, contextualizing where previous methods lie on the spectrum of probability of *feeling better*. The mean difference method, applied in the less severe PANDA sample, suggests an MCID of ~2 points or a SMD ~0.5–0.6, which is comparable to previous research [5,12]. We advocate for the ED50 to be used at each level of baseline severity as the mean will vary from study to study based on the severity of the sample. Indeed, when we include the more severe CoBalT sample, we find the mean of the ED50 estimates across patients yields somewhat higher MCID estimates (~3.5) in absolute terms. However, the averages of our proportionate changes (~20–30%) is very similar to previous estimates, as might be expected given that proportional change accounts for baseline severity [12]. The ED estimates suggest that previous methods in research settings appear to define the MCID as a probability of *feeling better* that lies somewhere between 25% and 50%. The 6- and 4-point PHQ-9 and GAD–7 estimates used in clinical practice appear to fall within 50% to 75% probability of response. [25,26]  Given the ambiguity of what can be defined as a clinically acceptable probability of response, the current method also affords flexibility to the user to determine which level of probability is appropriate in a given context.

Interestingly, patients with very low baseline severities do not appear to require an improvement in scores to have a 50% probability of *feeling better*. This initially appears to contrast our previous research, which used Bayesian hierarchical regression models and derived parameters to calculate the optimum sensitivity and specificity on a Receiver Operator Characteristics (ROC) curve and found patients with low baselines severity needed larger changes proportionate changes to *feel better* [12]. However, at very low baselines no change vs. a 1-point improvement translates into a large difference in proportionate change of 0% or 100%, respectively, for those with a baseline score of 1. Therefore, this seeming discrepancy is essentially two sides of the same coin, reflecting problems of estimation at the lower end of the scale which manifest differently according to the method used. This is supported by the observation that at low baseline severity the agreement between MCID and GRC responses appears lower [12]. It may be difficult for patients to discern a precise point at which they experience an improvement when there is such little scope to change in questionnaire scores. This suggests that the measures used may not be sufficiently sensitive for the lower ranges of severity, highlighting a need for further exploration of how to evaluate interventions in subclinical populations where conventional scales are at the limit of their operability.

Importantly, the present research also highlights a large range of MCID estimates which suggests that previous MCID estimates may be well suited for typical/average populations but may not capture the MCID across all patients equally well. Previous approaches provide an easy to implement guide but comes at a cost of 51% disagreement between the MCID and patient reports of improvement [12], [13]. The present approach is more specific, with ~ 23% better agreement, but at a cost of greater complexity to implement by providing an MCID for each level of baseline severity.

### 4.1 Strengths and limitations

The present study used data from two high-quality RCTs resulting in a large sample with clinically distinct populations, which is critical given that the MCID is baseline dependent. The GRC has clear face validity providing a useful patient-centered anchor [17].

The use of difference scores was a limitation as it ignores the measurement error; however, these effects are largely mitigated by the use of smoothing parameters in the statistical analysis. Furthermore, the GRC is subjective in nature - the concept of recovery is complex and unique to each patient. Clinical questionnaires commonly focus solely on symptoms. Responses to the GRC may incorporate wider (mental) health and psychosocial influences, such as comorbidities, life events, or quality of life, that may not be by captured by depression symptoms alone [27]. Further adjustment of predictors may improve the accuracy of the MCID estimates. However, these influences are likely to be wide and varied and would therefore require very large samples and could not be completed in the present analysis due to sample size limitations. It is also noteworthy that we assumed that the relationship between changes in outcome questionnaires and subjective improvements, was not affected by treatment. Future research could examine this relationship more closely and how it may be affected by different treatments and research design characteristics such as blinding. The secondary use of data resulted in further limitations. PANDA and CoBalT had different follow-up time points potentially resulting in time-dependent confounding. However, random effects were introduced to account for repeated measurements and the effects of time were not practically meaningful for estimating the MCID. The two studies also had differing levels of granularity of the GRC scales which meant we could only estimate the differences in mean change between those feeling *slightly better* and *the same* in PANDA. We used all of the data in our GAMM model, combining *same,* and *worse* into a single *not better* category to keep in line with our previous research. [5], [12] Our MCID estimates may be over-estimated as a consequence relative to methods which exclude those who feel worse (see Supplementary A). Although patients in both trials experienced depression and anxiety to varying degrees, the results indicate that greater changes are needed on the GAD–7 to feel better than the PHQ–9. Both studies recruited patients on the basis of depression as the primary problem. Changes in depression may have been perceived of greater relative importance, requiring smaller changes to *feel better*. As such, findings may not generalize to populations experiencing anxiety as their primary or only problem.

### 4.2 Implications

Despite the limitations, providing estimates to measure clinically meaningful change has important implications for research as well as clinical practice. In the analysis of results from clinical trials, the MCID could be applied to each patient within the treatment arms, allowing for comparisons between treatments on the number of patients who scored a change equal to or greater than the MCID. In a similar notion, the MCID could inform evaluations in clinical practice bringing greater face validity to experiences of symptomatic improvement in conceptualizations of clinical recovery. Equally, the within-subject change could be applied to examine between-treatment differences. Although the MCID might be relevant to superiority and equivalence trials, it may be particularly pertinent to non-inferiority trials where an alternative treatment is cheaper, less resource-intensive, or simpler to implement. Here, the MCID could be used to ascertain that the difference in treatment effects does not exceed the MCID; thereby, allowing for evaluations of cost-effectiveness that assure a newer or cheaper treatment is not of less benefit to patients. The ED50 MCID can inform sample size calculations by providing mean estimates of the expected change where at least 50% of patients would experience an improvement. They cannot inform the variance part of such calculations which will require wider considerations on the population studied. Baseline variability in outcome scores, however, is the major driver of patient heterogeneity and population level estimates of variance are easily obtainable.

### 4.3 Conclusion

The MCID contributes to our ability to assess clinically meaningful change rather than statistical significance alone. However, the research highlights the difficulty of calibrating patient experiences with structured questionnaires, such as the need to account for baseline severity. Here, we present an approach where the MCID is tailored to baseline severity to fully capture the entire spectrum of severity. Such approaches come at the cost of greater complexity but offer greater precision. The development and triangulation of different methods will advance our understanding of how abstract concepts can be defined mathematically and contextualize what different MCID approaches are measuring.

## Trial registration

Panda and CoBalT were registered with the Controlled Trials ISRCTN Registry: PANDA (ISRCTN84544741) and CoBalT (ISRCTN38231611).

## Acknowledgments

PANDA was funded by the National Institute for Health Research Programme Grant for Applied Research (RP-PG-0610-10048). CoBalT was funded by National Institute for Health Research Health Technology Assessment (Project Number 06/404/02). The research was supported by the NIHR Biomedical Research Centre at the University Hospitals Bristol NHS Foundation Trust, the University College London Hospital NHS Foundation Trust and the University of Bristol. The views expressed in this publication are those of the author(s) and not necessarily those of the Sponsor, NHS, NIHR or Department of Health, and Social Care. The funder had no role in the study design, data collection, data analysis, interpretation of data or writing of the present manuscript. We would like to thank all involved in participating, conducting or otherwise supporting both RCT and are grateful to all co-applicants of the RCT who were not involved in drafting the present manuscript.

## CRediT authorship contribution statement

**Clarissa Bauer-Staeb:** Conceptualization, Methodology, Formal analysis, Writing – original draft, Writing – review & editing, Visualization. **Daphne-Zacharenia Kounali:** Writing – review & editing. **Nicky J. Welton:** Writing – review & editing, Funding acquisition. **Emma Griffith:** Writing – review & editing, Supervision. **Nicola J. Wiles:** Writing – review & editing, Resources, Funding acquisition. **Glyn Lewis:** Writing – review & editing, Resources, Funding acquisition. **Julian J. Faraway:** Conceptualization, Methodology, Formal analysis, Writing – review & editing, Supervision. **Katherine S. Button:** Conceptualization, Methodology, Writing – review & editing, Supervision.

## Appendix Supplementary materials

Image, application 1
