# Supplementary material for: Effective dose 50 method as the minimal clinically important difference: Evidence from depression trials
Source: J Clin Epidemiol. 2021 Sep;137:200–8. doi: 10.1016/j.jclinepi.2021.04.002 (PMC8485844; doi:10.1016/j.jclinepi.2021.04.002)
Supplement: Supplementary file 1 [file mmc1.pdf]

SUPPLEMENTARY MATERIAL

1. Supplementary Material A - Justification of Methodology

1.1 Dealing with baseline dependency

Baseline dependency is a well-established problem – the change needed to feel better varies according to baseline severity, with patients who have more symptoms commonly requiring greater changes to experience a subjective improvement.[1-3] Various methods to address this problem. The most prominent methods include effect sizes, statistical control, proportionate change, and MCID categories.[1] Mean change methods broadly-speaking encompasses approaches that examine the mean change at different levels of the GRC. That is, two of the originally developed methods include examining the mean change amongst those who *feel slightly better* or calculating the difference in mean change of those responding feeling *slightly better* and *feeling about the same*.[4,5] This method does not take baseline dependency into account. To account for baseline dependency, effect sizes can be estimated.[6,7] While effect sizes are useful for comparative purposes, they have been criticised for providing little clinical information and being difficult to interpret.[8] Statistical control can be implemented in models to reduce the effect of baseline severity.[1] However, Copay et al. reports that because extreme scores are assumed to be a result of error/chance, the true variation is masked despite the fact that medical patients might be expected to have higher symptom scores.[1] Proportionate change is the percentage of how much someone's symptoms change relative to their baseline score. While this approach is beneficial as it allows for comparisons between measures, it may increase the association with baseline scores when patients with high symptoms have small change.[1] A further approach is to provide multiple MCID estimates for categories of patients, which are grouped based on the certain levels of the measure.[1] These can sometimes create somewhat arbitrary groups and reduce the benefit of one MCID figure.[1] Previous analyses have shown that using proportionate scores in depression and anxiety provide a good rule of thumb for those with moderately-severe symptoms, but do not fully account for baseline dependency at all ranges and may additionally require categorization of the MCID based on baseline severity categories (mild, moderate, severe).[2,3] Our previous and present analyses additionally show that adjustment for baseline severity, either as an interaction term in linear models of by using percent change, is insufficient to fully account for baseline severity across the scale.[2] Our argument is that if it is not possible to fully capture baseline dependency and produce one universal MCID with the methods above, it may be worthwhile having a more detailed and precise approach that, by definition, fully captures baseline severity to be used alongside the existing rule-of-thumb.

1.2 Modelling Approach

The MCID is a concept rather than being mathematically defined. As such, multiple methods have been proposed to estimate the MCID. Some of the most common methods include mean change, linear regression, and Receiver Operator Characteristics (ROC) curve.[1,7,9] A comprehensive review of MCID approaches can be found elsewhere.[1,7,9] As above, mean change methods don't account for baseline dependency and standardised differs have been criticised for providing little clinical information and being difficult to interpret.[4-9] Using linear regression allows the mean changes to be adjusted for baseline severity to account for baseline dependency. [1,7,9,10] However, previous research and the current analyses show that baseline adjustment is insufficient to fully account for baseline dependency in depression and anxiety across the spectrum.[2,3] ROC curves identify the point of optimal sensitivity and specificity between two groups of GRC responses (i.e., those who *feel better* vs. those who do *not feel better*) to denote the MCID.[1,2,9,10] However, they have the limitation that this estimate can be unstable and subject to changes in the ROC curve (and thus the relative balance of sensitivity/specificity) following the addition/deletion of a few data points.

We propose adopting the ED50 as a new means of estimating the MCID. The ED50 is frequently used in drug safety and pharmacotherapy research to identify the minimum effective therapeutic dose.[11] An example of an application is the prescription of drugs, where the ED50 is used as a guideline for clinicians to identify the smallest effective dose of medication.[11] For the purposes of this analysis, the MCID is defined as the changes in scores associated with a 50% probability of *feeling better.* The ED50 has clear face validity as a MCID metric as it marks the threshold where patients are slightly more likely to feel *better* than *not*. The ED50 is based on a model derived from all data and therefore not as susceptible to the limitations of the ROC analysis and mean change methods. Using this approach, we further address the problem of baseline dependency above and beyond covariate adjustment in GLM. By incorporating baseline severity in the GAMM model additively we provide an MCID for each level of baseline severity. Thus, addressing the limitation that covariate adjustment alone does not appear to fully account for baseline dependency in depression and anxiety. Using this method also allows for the identification of any important difference (i.e., ED25 and ED75) to examine the probabilities associated with different changes, which provides a granularity that other approaches do not. This provides flexibility to the end user to identify and select the amount of certainty in treatment response.

1.3 Data and statistical model decisions

1.3a Inclusion of all GRC responses

A common approach to estimating the MCID is to examine the mean change amongst those *who feel slightly* better or to examine the difference in mean change of those responding feeling *slightly better* and *feeling about the same* to find the minimal clinically important difference.[4,5] CoBalT patients were asked how they felt in comparison to the last assessment to which they could respond: "I feel better", "I feel about the same", and "I feel worse".[12] In PANDA, patients were asked how they felt compared to when they were last seen at all time points, with fixed responses entailing: "I feel a lot better", "I feel slightly better", "I feel about the same", "I feel slightly worse", and "I feel a lot worse".[13] As such, only one of the RCTs contained a more fine-grained breakdown and we were limited by the data available. This approach to estimating mean change is useful, but it has the limitation of throwing away a lot of data where only one or two of several fine-grained GRC categories are of interest, or when the categories are limited (as with CoBalT) the estimates can be inflated by inclusion of those people who felt very much better.

When estimating from statistical models (GLM or GAMM) it is preferrable to include all observations to reduce bias and increase the precision of the model. Examining only subgroups of patients can lead to erroneous results.[14] As such, we included all GRC responses in the present analysis. The present analysis nonetheless examines the minimal point as it is modelling the probability of feeling better by baseline severity and change in symptoms, rather than looking at the mean differences stratified by the GRC. From this model, we estimate the MCID as a threshold (a lower bound, if you will) of 50% chance or greater of feeling better. Unlike the categorical mean change approach this threshold is relatively robust to the inclusion of those with a wide range of GRC ratings.

1.3b Exclusion of time as a model effect

We found a statistically significant effect of time on the proportion of people feeling better at follow-up 1. However, we are interested in MCID estimates, and when we examined the effect of time on the ED50 (MCID estimates) we found marginal differences as a result of time, that were of little practical importance to the MCID estimates. We therefore excluded time from the final model for pragmatic purposes; future users will want to select a MCID without having to decide which follow-up period is closest to theirs. Unless there is strong evidence that time makes a practically significant difference to the MCID there is little benefit of adding time for the end-user.

1.3c Pooling of studies and exclusion of study as a model term

We pooled data from two RCTs. This has the benefit of higher precision as there are more observations per level of baseline severity. We found a statistically significant effect of study on the probability of feeling better driven by differing baseline severities at follow-up 1, with PANDA having fewer observations at the very high end of scores, and CoBalT fewer lower scores.[10,11] As such, MCID estimates for the low end of scores from CoBalT at follow-up 1 alone will be unreliable. The opposite is true for PANDA. The effect of study disappeared after follow-up 1, further suggesting that the initial difference is a result of the different baseline characteristics of the RCTs and will therefore have little practical importance to the MCID estimates. Pooled data is preferable as it provides a greater coverage of baseline scores at time point 1 and the model produces a weighted average where most of the weight comes from one study. Similar to the covariate of time, the effects of study on the MCID estimates were of little practical importance. We therefore exclude study from the final model. This is advantageous because future users will want to select an MCID for their study without having to decide whether it is more like one of the two studies – the result is more generalisable in this way.

1.3d Inclusion of treatment and control groups

We include both treatment and control groups in the present analyses for the purposes of generalisability. Our primary aim is to identify a change in scores that is noticeable to patients, but we are agnostic to how this change is produced. We assume a stability in the relationship between the changes in symptoms and the GRC that does not vary by treatment. We have no reason to believe that different treatments require a different MCID, i.e., if a patient changes a given amount on the PHQ-9 they should be as likely to notice this difference regardless of whether it was brought about by SSRIs, CBT or placebo/natural recovery. From our perspective (for the purposes of this analysis), the treatment is simply a means of inducing change.

1.3e No further covariate adjustment

While the adjustment of various other covariates is technically possible, we are unable to implement them in the current analyses. Firstly, there is a sample size consideration as we are stratifying by each level of baseline severity. This analysis would require much larger sample sizes for the adjustment of additional covariates, which would only be feasible through the analysis of electronic healthcare records or pooling of a very large number of clinical trials. Unfortunately, we are limited by the data available to us. However, there are also pragmatic issues with further covariate adjustment. In order to produce generic MCID estimates, the covariates would have to be fixed at certain points to estimate the ED50, introducing an array of assumptions that may not hold across all patients. Alternately, an MCID can be estimated for each patient individually. However, this would firstly require that data to be available, creating the burden of additional data collection on patients and clinicians/researchers. This may be difficult in clinical practice due to time constraints but also in clinical research where these measures may otherwise not be of interest. Secondly, the calculation would be too extensive to print in any format and would require an online resource.

REFERENCES

[1] Copay AG, Subach BR, Glassman SD, Polly Jr DW, Schuler TC. Understanding the minimum clinically important difference: a review of concepts and methods. The Spine Journal. 2007 Sep 1;7(5):541-6. https://doi.org/10.1016/j.spinee.2007.01.008

[2] Button KS, Kounali D, Thomas L, Wiles NJ, Peters TJ, Welton NJ, Ades AE, Lewis G. Minimal clinically important difference on the Beck Depression Inventory-II according to the patient's perspective. Psychol Med. 2015 Nov;45(15):3269-79. http://doi.org/10.1017/S0033291715001270

[3] Kounali D, Button KS, Lewis G, Gilbody S, Kessler D, Araya R, Duffy L, Lanham P, Peters TJ, Wiles N, Lewis G. How Much Change is Enough? Evidence from a longitudinal study on depression in UK Primary Care. Psychol Med. 2020 Nov 3:1-8. doi:10.1017/S0033291720003700

[4] Jaeschke R, Singer J, Guyatt GH. Measurement of health status: ascertaining the minimal clinically important difference. Control Clin Trials. 1989 Dec 1;10(4):407-15. https://doi.org/10.1016/0197-2456(89)90005-6

[5] Redelmeier DA, Lorig K. Assessing the clinical importance of symptomatic improvements: an illustration in rheumatology. Arch Intern Med. 1993 Jun 14;153(11):1337-42.

[6] Borenstein M. Effect sizes for continuous data. In: Cooper H, Hedges LV, Valentine JC (Eds.): The Handbook of research synthesis and meta-analysis. Russell Sage Foundation, New York 2009 (2nd Ed.):12:222-236.

[7] Angst F, Aeschlimann A, Angst J. The minimal clinically important difference raised the significance of outcome effects above the statistical level, with methodological implications for future studies. J Clin Epidemiol. 2017 Feb 1;82:128-36. https://doi.org/10.1016/j.jclinepi.2016.11.016

[8] Cuijpers P, Karyotaki E, Weitz E, Andersson G, Hollon SD, van Straten A. The effects of psychotherapies for major depression in adults on remission, recovery and improvement: a meta-analysis. J Affect Disord. 2014 Apr 20;159:118-26. https://doi.org/10.1016/j.jad.2014.02.026

[9] Mills KA, Naylor JM, Eyles JP, Roos EM, Hunter DJ. Examining the minimal important difference of patient-reported outcome measures for individuals with knee osteoarthritis: a model using the knee injury and osteoarthritis outcome score. J Rheumatol. 2016 Feb 1;43(2):395-404. https://doi.org/10.3899/jrheum.150398

[10] Copay AG, Eyberg B, Chung AS, Zurcher KS, Chutkan N, Spangehl MJ. Minimum clinically important difference: current trends in the orthopaedic literature, part II: lower extremity: a systematic review. JBJS reviews. 2018 Sep 1;6(9):e2. DOI: 10.2106/JBJS.RVW.17.00160

[11] Dimmitt S, Stampfer H, Martin JH. When less is more–efficacy with less toxicity at the ED50. Br J Clin Pharmacol. 2017 Jul;83(7):1365. https://doi.org/10.1111/bcp.13281

[12] Wiles N, Thomas L, Abel A, Ridgway N, Turner N, Campbell J, Garland A, Hollinghurst S, Jerrom B, Kessler D, Kuyken W. Cognitive behavioural therapy as an adjunct to pharmacotherapy for primary care based patients with treatment resistant depression: results of the CoBalT randomised controlled trial. Lancet. 2013 Feb 2;381(9864):375-84. https://doi.org/10.1016/S0140-6736(12)61552-9

[13] Lewis G, Duffy L, Ades A, Amos R, Araya R, Brabyn S, Button KS, Churchill R, Derrick C, Dowrick C, Gilbody S. The clinical effectiveness of sertraline in primary care and the role of depression severity and duration (PANDA): a pragmatic, double-blind, placebo-controlled

randomised trial. Lancet Psychiatry. 2019 Nov 1;6(11):903-14. https://doi.org/10.1016/S2215-0366(19)30366-9.

[14] McGlothlin AE, Lewis RJ. Minimal clinically important difference: defining what really matters to patients. JAMA. 2014 Oct 1;312(13):1342-3. doi:10.1001/jama.2014.13128

2. Supplementary Material B – Correlation between the Global Rating of Change and Change in Questionnaire

Supplementary Material B. Spearman rank correlation coefficients between change in symptoms and the categorical Global Rating of Change, stratified by study and follow-up

		**Baseline to Follow-up 1**	**Follow-up 1 to Follow-up 2**	**Follow-up 2 to Follow-up 3**	**Follow-up 3 to Follow-up 4**
**Patient Health Questionnaire -9**	*PANDA*	-0.41	-0.50	-0.43	-
	CoBalT	-0.46	-0.43	-0.38	-0.32
**Generalised Anxiety Disorder Scale-7**	*PANDA*	-0.37	-0.43	-0.36	-
	*CoBalT**	-	-0.52	-	-0.41

*Generalised Anxiety Disorder Scale-7 data was not collected at follow-up one and three. Change scores are derived from previous follow-up.

Data reported for patients with complete Global Rating of Change and each respective outcome questionnaires.

The Global Rating of Change for Panda contains 5 categories and 3 categories in CoBalT.

3. Supplementary Material C – Model Summaries of the Generalised Additive Mixed Models

Supplementary Material C. Summary of the Generalised Additive Mixed Models

Model	*Observations*	*Adjusted r2*	*Deviance explained*	*UBRE*	*AIC*	*Variable(s)*	*P-value*
Patient Health Questionnaire -9 (1)	3205	0.468	46.80	0.050	3366	Change x Baseline severity	<0.001
						ID	<0.001
Patient Health Questionnaire -9 (2)		0.468	46.60	0.045	3350	Change x Baseline severity	<0.001
						ID	<0.001
						Period 2	0.002
						Period 3	0.111
						Period 4	0.406
						Study: CoBalT	0.001
Generalised Anxiety Disorder Scale-7 (1)	2415	0.390	38.80	0.108	2676	Change x Baseline severity	<0.001
						ID	<0.001
Generalised Anxiety Disorder Scale-7 (2)		0.404	40.00	0.097	2649	Change x Baseline severity	<0.001
						ID	<0.001
						Period 2	<0.001
						Period 3	<0.001
						Study: CoBalT	0.042

UBRE- Un-Biased Risk Estimator; AIC - Akaike Information Criterion; PHQ-9 - Patient Health Questionnaire - 9-item; GAD-7 - Generalised Anxiety Disorder 7-item

4. Supplementary Material D – 95% Confidence Intervals of Generalised Additive Mixed Models

The following graphs present slices through smooth surface plots at the mild, moderate, and severe thresholds for each outcome questionnaire. The predicted values are presented on the logit scale to assess variability at each level of change, with corresponding 95% confidence intervals. Limits were set the maximum obtainable change for each level of baseline severity.

The confidence intervals widen slightly towards the extreme ends of change, particularly when baseline severity is low and extreme reduction (deteriorations) take place as it was rare for patients with such mild symptoms to deteriorate drastically. This is unlikely to have an impact on the ED50 estimates, as they are focused on positive changes above 0, where the confidence intervals are visibly narrower.

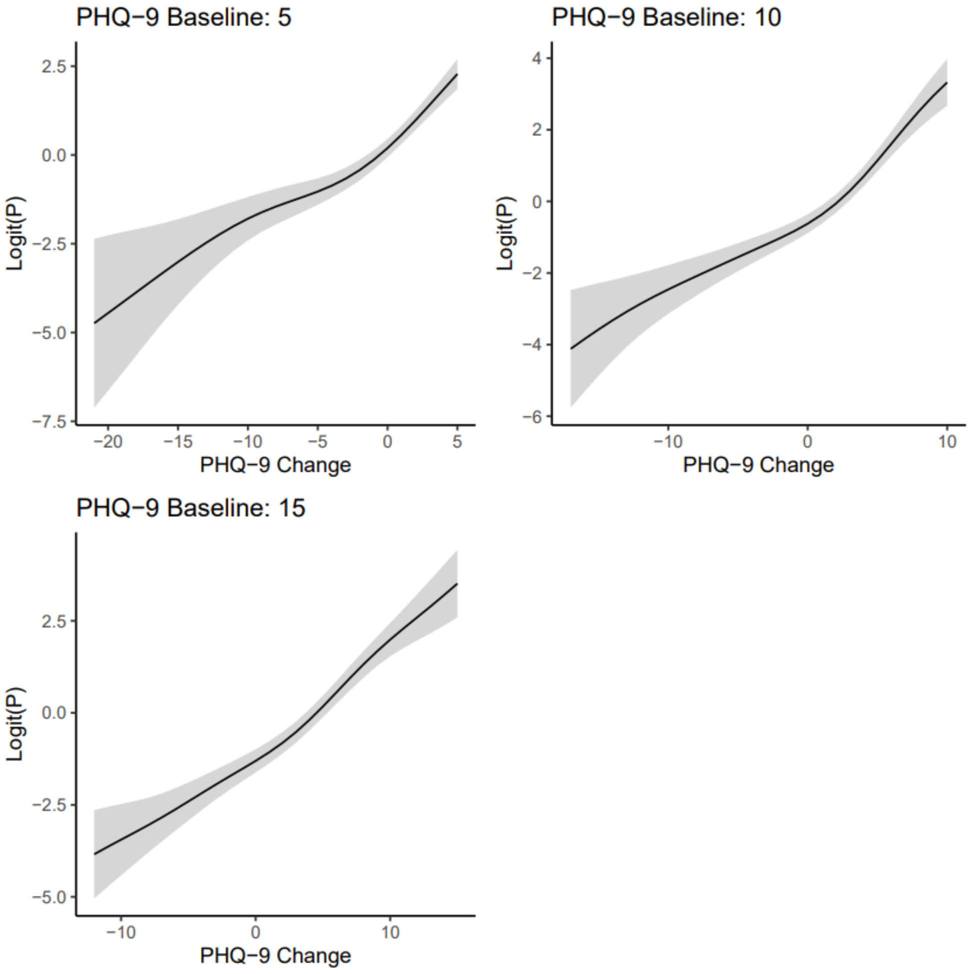

Supplementary Material D.1. Slices of the smoothed surface plots with 95% confidence intervals for the Patient Health Questionnaire -9

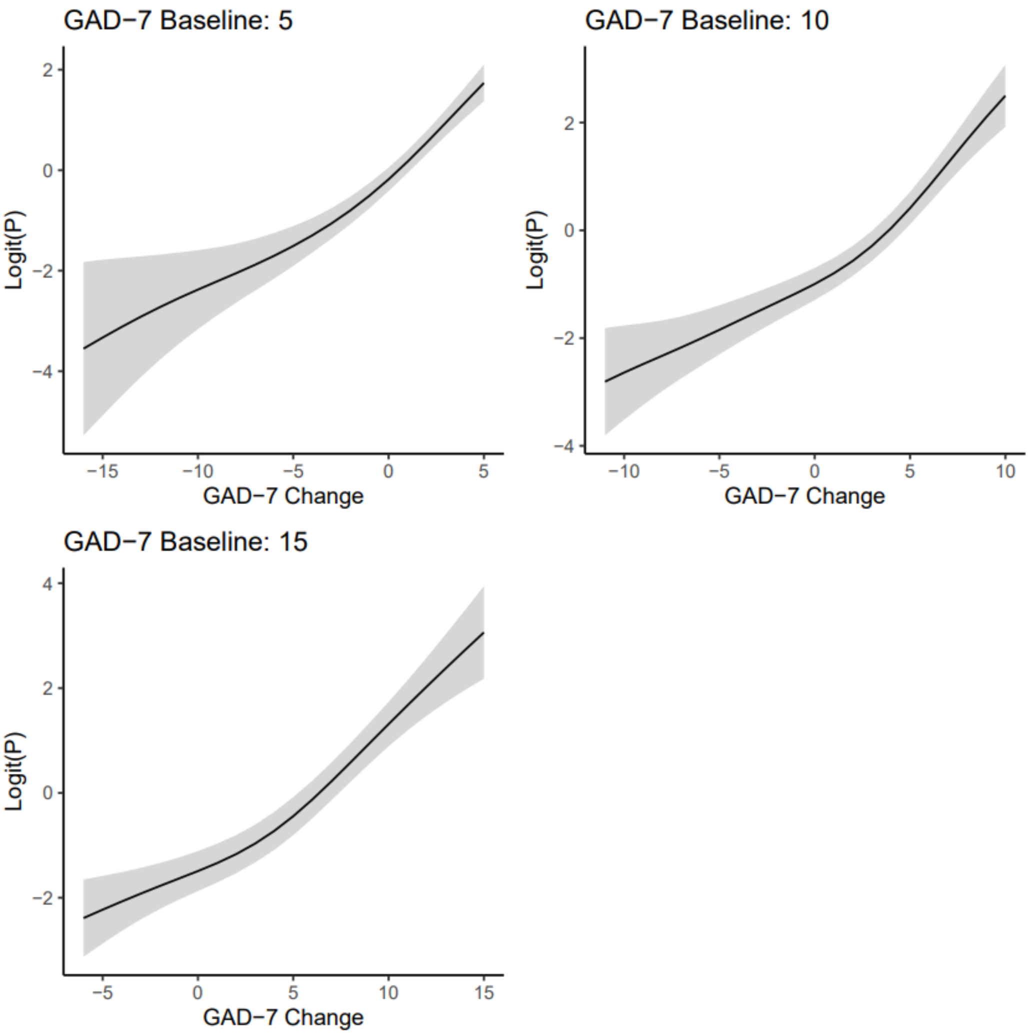

Supplementary Material D.2. Slices of the smoothed surface plots with 95% confidence intervals for the Generalised Anxiety Disorder Scale-7
